# Anti-Obesity Therapeutic Targets Studied In Silico and In Vivo: A Systematic Review

**DOI:** 10.3390/ijms25094699

**Published:** 2024-04-25

**Authors:** Wendjilla F. de Medeiros, Ana Francisca T. Gomes, Ana Júlia F. C. Aguiar, Jaluza Luana C. de Queiroz, Ingrid Wilza L. Bezerra, Juliana Kelly da Silva-Maia, Grasiela Piuvezam, Ana Heloneida de A. Morais

**Affiliations:** 1 Nutrition Postgraduate Program, Center for Health Sciences, Federal University of Rio Grande do Norte, Natal 59078-900, Brazil; wendjillanutri@gmail.com (W.F.d.M.); aft.gomes00@gmail.com (A.F.T.G.); ingrid.bezerra@ufrn.br (I.W.L.B.); juliana.maia@ufrn.br (J.K.d.S.-M.); 2 Biochemistry and Molecular Biology Postgraduate Program, Biosciences Center, Federal University of Rio Grande do Norte, Natal 59078-970, Brazil; anajulianutri@gmail.com (A.J.F.C.A.); luh.queiroz.nutri@gmail.com (J.L.C.d.Q.); 3 Department of Nutrition, Center for Health Sciences, Federal University of Rio Grande do Norte, Natal 59078-900, Brazil; 4 Public Health Postgraduate Program, Center for Health Sciences, Federal University of Rio Grande do Norte, Natal 59078-400, Brazil; gpiuvezam@yahoo.com.br; 5 Public Health Department, Federal University of Rio Grande do Norte, Natal 59078-900, Brazil

**Keywords:** computer simulation, molecular dynamics simulation, molecular docking simulation, peptides, molecular conformation, obesity

## Abstract

In the age of information technology and the additional computational search tools and software available, this systematic review aimed to identify potential therapeutic targets for obesity, evaluated in silico and subsequently validated in vivo. The systematic review was initially guided by the research question “What therapeutic targets have been used in in silico analysis for the treatment of obesity?” and structured based on the acronym PECo (P, problem; E, exposure; Co, context). The systematic review protocol was formulated and registered in PROSPERO (CRD42022353808) in accordance with the Preferred Reporting Items Checklist for Systematic Review and Meta-Analysis Protocols (PRISMA-P), and the PRISMA was followed for the systematic review. The studies were selected according to the eligibility criteria, aligned with PECo, in the following databases: PubMed, ScienceDirect, Scopus, Web of Science, BVS, and EMBASE. The search strategy yielded 1142 articles, from which, based on the evaluation criteria, 12 were included in the systematic review. Only seven these articles allowed the identification of both in silico and in vivo reassessed therapeutic targets. Among these targets, five were exclusively experimental, one was exclusively theoretical, and one of the targets presented an experimental portion and a portion obtained by modeling. The predominant methodology used was molecular docking and the most studied target was Human Pancreatic Lipase (HPL) (n = 4). The lack of methodological details resulted in more than 50% of the papers being categorized with an “unclear risk of bias” across eight out of the eleven evaluated criteria. From the current systematic review, it seems evident that integrating in silico methodologies into studies of potential drug targets for the exploration of new therapeutic agents provides an important tool, given the ongoing challenges in controlling obesity.

## 1. Introduction

Improving the effectiveness of strategies for the prevention, control, and treatment of obesity has been placed as a priority in the context of global public health [1]. Worldwide, there is a trend of increasing numbers of adults diagnosed with obesity. It is estimated that in 2020, the number of cases reached 810 million, with projections indicating that by 2035 this number will reach 1.53 billion [2].

Due to its multifactorial etiology, the development of obesity is associated with complex interactions between biopsychosocial factors, such as behavioral, genetic, and environmental factors [3]. The implications of obesity reverberate significantly across individuals, society, and the economy, given its nexus with premature mortality [4] and contribution to the increased prevalence of comorbidities, such as type 2 diabetes mellitus (T2DM) [5], cardiovascular diseases (CVD) [6], and some types of cancer [7].

Achieving a reduction in body weight by 5% or more already promotes substantial benefits to individuals with obesity, including an improvement in glycemic levels, lipid profile, depression, and mobility, a reduction in hepatic steatosis, and an improvement in general quality of life [8]. The non-surgical pillars currently available for the obesity management are adherence to healthy eating, physical activity, psychological interventions, and pharmacotherapy [9]. However, such therapeutic interventions have limitations, such as low adherence and difficulty maintaining weight loss, which compromise long-term success [1].

Across history, numerous pharmacological agents have been sanctioned for obesity treatment, and subsequently withdrawn from use owing to their untenable adverse effects. For instance, Lorcaserin and amphetamine were discontinued due to an elevated risk of carcinogenesis and the potential for inducing chemical dependence, respectively. [8]. In contrast, numerous substances are undergoing tested for the treatment of obesity, such as survodutide, mazdutide, and orforglipron, which not only demonstrates an intense search for therapeutic success but also highlights a gap [10,11,12]. Unfortunately, only a limited number of drugs satisfy the fundamental criteria of efficacy and safety necessary for obtaining approval [13].

However, the knowledge about obesity’s pathophysiology has advanced in recent years, leading to the expansion of potential therapeutic targets for interventions [13], such as the leptin receptor and Pancreatic Lipase (PL) [14,15]. Currently, anti-obesogenic agents with central mechanisms of action exert their effects on elements within the central nervous system (CNS) to promote the regulation of satiety and fullness, while peripherally acting substances target physiological components involved in digestion, metabolism, and/or lipogenesis [16].

Simultaneously, a rapid advance can be seen within bioinformatics, which proves to be a strong ally by enabling, through simulations, the verification of the possible interaction between these therapeutic targets and the new substances of interest [17]. In recent years, the range of experimentally resolved 3D biostructures has been cataloged within databases. This expansion has been accompanied by advancements in tools dedicated to the theoretical prediction of protein structure [18].

The term computer-assisted drug design (CADD) integrates a set of computational methods that can be applied to optimize the search for new drug candidates, anticipating unfavorable outcomes and minimizing the number of new agents to be biochemically tested. CADD can be grouped into two subdivisions, the first of which consists of “structure-based design” (SBDD), where the selection of agents is based on the previously elucidated structure of the target, either experimentally or via computational modeling. Alternatively, “ligand-based drug design” (LBDD) is based on the structures of ligands known for their integration with targets, for the design of new agents, being an option, for example, when the structure of the target is not yet known [19].

Among the methodologies used in SBDD, the most commonly used are structure-based virtual screening (SBVS), docking, and molecular dynamics (MD) [20]. Molecular docking is a computational method used to predict the binding mode between a candidate agent and the biological target of interest, obtaining information regarding favorable orientations, binding conformations, and binding affinity [21]. In the SBVS technique, a funnel method is employed wherein libraries of chemical structures of interest are automatically evaluated, and the agents with the best binding scores to the biological target (receptor) are selected for subsequent steps [22].

MD simulations employs the equations of motion to calculate the temporal positions and velocities of the system’s atoms during interaction, generating information about the movement and interaction of molecules [23]. Therefore, it represents one of the possible computational approaches used in the development of new drugs, allowing the evaluation of conformational changes and ligand–target interactions. During simulations, it is also possible to incorporate different experimental conditions, such as variations in pH and temperature [24,25].

These advancements enhance studies of bioinformatics applied to healthcare across diverse areas, as evidenced by analyses of drug approvals by the Food and Drug Administration (FDA). Among the drugs that were approved between 2010 and 2016, the discovery and development stages of 210 drugs were facilitated through the utilization of three-dimensional structures deposited in databases [26].

Coadjuvant therapeutic strategies for the treatment of obesity and its comorbidities have been developed. Among these are bioactive compounds, which have shown potential health benefits through activities such as the regulation of gene expression in adipose tissue, and the modulation of adipokine and hormone secretion [27]. Pharmacological constraints, encompassing safety, maintenance of weight loss, and efficacy of current drugs underscore the necessity for research advances [28]. Thus, given the limited capacity of current tools, it is essential to continue seeking therapeutic targets and substances with anti-obesity potential, contributing to overcoming the undeniable global difficulty in controlling the increase in cases and consequently minimizing their socioeconomic burden.

To the authors knowledge, to date, no systematic reviews have been executed to explore therapeutic targets of obesity through the combined effort of in silico coupled to in vivo validation. Thus, this systematic review aims to ascertain which anti-obesity targets have been studied using docking and/or molecular dynamics (MD) techniques with subsequent in vivo validation.

## 2. Methods

### 2.1. Protocol and Registration

Before conducting the systematic review, the protocol was registered on the International Prospective Platform for Systematic Reviews (PROSPERO), following the Preferred Reporting Items Checklist for Systematic Review and Meta-Analysis Protocols (PRISMA-P) on 26 August 2022, under the number CRD42022353808 from the following access link: https://www.crd.york.ac.uk/prospero/display_record.php?RecordID=353808, (accessed on 30 December 2022) and published in a scientific journal [29]. Thus, the systematic review proceeded as planned, but some adaptations were necessary and are included in the PROSPERO record. This study is exempt from evaluation by the Research Ethics Committee (RES).

### 2.2. Search Question

The research question “What are the therapeutic targets used in the treatment of obesity in in silico studies?” was structured using the acronym PECo (P, problem; E, exposure; Co, context) (Table 1).

### 2.3. Inclusion Criteria

Original computer simulation studies that performed MD studies and/or molecular docking with interactions between different substances and human therapeutic targets for the treatment of obesity were included, selecting studies whose results were validated with in vivo studies.

### 2.4. Exclusion Criteria

Studies exclusively in vivo, studies exclusively in vitro, preprints, data in brief, guidelines, review articles, theses, dissertations, letters, conference abstracts, gray literature, and studies without in vivo experimental validation were excluded. Bioinformatics studies that used non-human experimental or theoretical structures or with mutations, experimental structures without structure code, targets obtained by modeling without sequence code or without the respective templates, and studies that did not perform MD and/or molecular docking were also excluded, as well as studies focusing on other comorbidities and/or conditions.

### 2.5. Search Strategy

The bibliographic search was executed on 21 July 2023, using the following electronic databases: PubMed; ScienceDirect; Scopus; Web of Science; Virtual Health Library (VHL) and EMBASE. Additionally, a manual search for studies that may not have been captured by the search strategies employed was also carried out.

The development of the search strategy, designed for heightened sensitivity, began with the selection of terms in a controlled vocabulary. Based on the research question, MeSH (Medical Subject Heading) health descriptors in Medline along with their corresponding entry terms were selected. The terms selected for constructing the search strategy were “target”, “therapeutic target”, “treatment”, “obesity”, “obese” “in silico”, “computer simulation”, “molecular dynamics simulation”, “molecular dynamics”, “molecular docking simulation”, and “molecular docking”. It is important to emphasize that the terms “target” and “therapeutic target”, though not part of the controlled vocabulary, were imperative for retrieving pertinent studies in alignment with the proposed question.

No limitations regarding publication timeframe or language were applied to the search. The terms selected to compose the search strategy were combined with the Boolean logical operators of intersection “OR” and addition “AND”. Additionally, truncation symbols were added to cover a wider range of words.

To identify the most appropriate search equation for the selection of articles, based on the research question, several strategies were tested, compared, and refined. This process took into account the distinctive attributes of various databases. The strategies developed for each database are shown in Table 2.

### 2.6. Data Selection and Extraction

After searching for articles in the selected databases, the studies obtained were exported to the Rayyan software (version 0.1.0) [30]. The process of selecting and extracting data was carried out by two independent reviewers, and any discrepancies were solved by a third reviewer.

The Rayyan tool enabled the management of the articles and exclusion of duplicates. It was used during the initial phase of study selection, during which reviewers assessed titles and abstracts. Sequentially, articles selected in the first stage processed to the full reading phase, where inclusion or exclusion was applied based on pre-defined criteria. The excluded articles and their justifications were recorded. The summary of the articles selection and exclusion process was presented through the flowchart for selection of articles, in accordance with the Preferred Reporting Items Checklist for Systematic Review and Meta-Analysis (PRISMA).

The selected articles for inclusion in the study underwent data extraction conducted by two independent reviewers. The extracted data encompassed the following attributes: authors, language, model (in silico), technique used (docking and MD), amino acids with greater interaction, and main outcomes (i.e., type of therapeutic agent, in vivo effects, and potential applications). The collected data from the selected articles were tabulated into a pre-defined table using the Microsoft Excel version 2016 software program.

For studies where full electronic access was not possible, either due to unavailability or restricted access requiring payment, the respective authors were contacted via email (max. 2 attempts) in an effort to obtain the studies.

### 2.7. Data Analysis and Synthesis

The obtained data were synthesized using a narrative approach. Descriptive tables were created to display the characteristics of the studies, along with information about outcomes and research protocols. These details were condensed and organized based on their respective therapeutic targets. The organization of the references was managed through the Mendeley software program (version 1.19.8). Meta-analysis was not applied due to the types of selected studies and the variety of outcomes found, the approach being more focused on descriptive aspects.

### 2.8. Assessment of the Risk of Bias in the Included Studies

The assessment of bias risk was conducted independently by two researchers, and in cases of disagreements, a third evaluator participated to reach a consensus. The assessment of the risk of bias in the studies followed the adapted checklist developed by Taldaev and collaborators [31], as there is no universally standardized tool in the literature that encompasses molecular dynamics and/or docking studies. The evaluators were trained and calibrated for the application of the tools.

In order to contemplate the studies, three adaptations were made to the tool. One adaptation in the topic refers to the verification of the results of the docking by in vitro studies, including the insertion of the “in vivo” validation option. Two adjustments were implemented in relation to the topic “target selection”, both referring to the methods employed for obtaining the structures. Along with the other options with a high risk of bias, the “homology modeling” option was included, since, despite its limitations, it can be used to determine 3D structures that have not yet been experimentally resolved. The second change within this topic involves reclassifying the “X-ray crystallography” technique. originally classified with a “high risk of bias”, it has now been reclassified as having a “low risk of bias”. The alteration is because X-ray crystallography is an experimental method that is especially relevant in the study of large biomolecules, allowing the experimental elucidation of protein structures at high atomic resolution (Table S1).

## 3. Results and Discussion

### 3.1. Articles Included in Search

**Figure 1:**
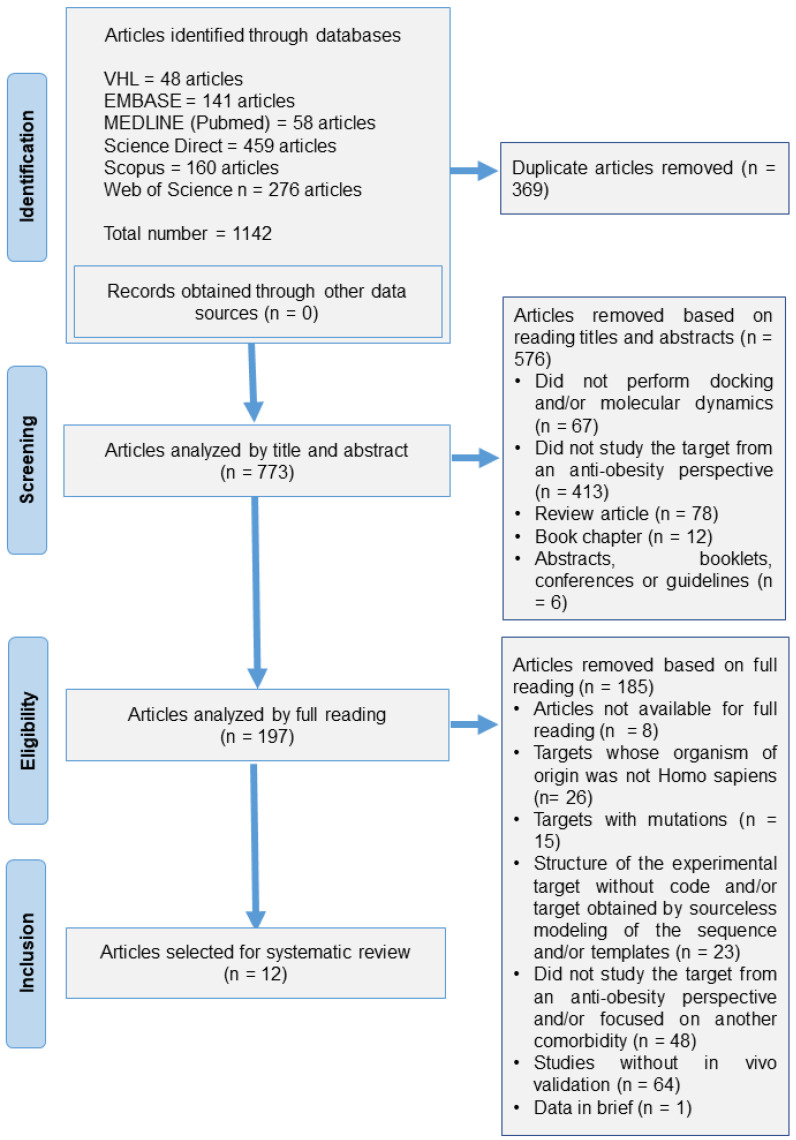
Article selection flowchart adapted from Preferred Reporting Items for Systematic Reviews (PRISMA).

Initially, the search across electronic databases yielded a total of 1142 publications, resulting in the identification of seven therapeutic targets (Table 3) from the 12 studies that fully met the research inclusion criteria (Figure 1). The 12 studies included were published in English between 2008 and 2023.

Within these studies, information regarding seven different in silico therapeutic targets adhering to predefined criteria were identified and extracted. These targets include the leptin receptor (LEPR), protein tyrosine phosphatase 1B (PTP1B), the fat mass and obesity-associated protein (FTO), human LP, the type 1 cannabinoid receptor (CB1), CD36 receptor, and Acetyl-CoA carboxylase (ACC). Notably, among the studies selected for systematic review, only one carried out MD simulations, predominantly molecular docking as methodology (Table 3).

In total, 12 studies met all the inclusion criteria, thus proceeding to the steps of extraction, synthesis, data analysis, and the assessment of the risk of bias (Table 3).

### 3.2. Risk of Bias Assessment

**Figure 2:**
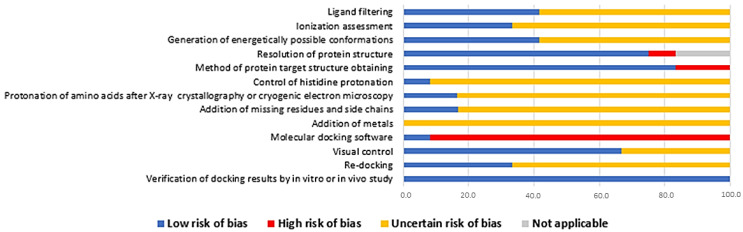
Graphical representation of the risk of bias of studies selected for systematic review (n = 12). Note: tool adapted from Taldaev and collaborators [31].

The risk of bias in the studies was assessed as proposed by the checklist adapted from Taldaev et al. [31] (Figure 2). Regarding the selection of ligands, only 41.7% of the studies clearly indicated the presence of filtering steps, 33.3% explicitly mentioned the ionization of the ligands, and 41.7% indicated the generation of energetically favorable conformations for these selected ligands. In terms of the resolution of the experimental structures of the targets, 75% did not exceed 2.5 Å, resulting in a low risk of bias. Additionally, only two studies (16.7%) were classified as having a high risk of bias regarding the method of structure obtaining, as their 3D structure was theoretically derived.

Topics associated with target optimization had the highest number of studies categorized with an “uncertain risk of bias”. In relation to aspects such as the control of histidine protonation, amino acid protonation, the addition of residues and absent side chains, and the addition of metallic ions, the percentages of studies classified with an uncertain risk of bias were 91.7%, 83.3%, 83.3% and 100%, respectively.

Regarding the software used for the docking study, only 8.3% of the studies received the “low risk of bias” classification. The last point was related to the evaluation of the results, with 66.7% of the studies conducting visual control, thereby receiving the low-risk classification. However, only 33.3% reported re-docking as part of their studies. In the context of the last evaluated aspect, considering it an option for reassessment and validation with an in vivo study, all studies were classified as “low risk of bias” in this aspect.

The analyzed studies underscore the necessity to enhance the level of methodological intricacy in bioinformatics research dedicated to deriving anti-obesity agents from obesity-related therapeutic targets. The assessment of the risk of bias shows that in eight aspects, more than 50% of the studies were classified as having “unclear risk of bias” due to the insufficient methodological detail. The omission of such information compromises the evaluation of the quality of studies and reproducibility by the scientific community.

Accordingly, it is essential to properly select and prepare the targets to obtain quality results in methodologies based on the structure of the target, such as the development of pharmacophoric models. It is essential to be careful with aspects such as the appropriate protonation state, insertion of hydrogen atoms that may be absent in the original structure, possible residues or atoms missing in the structure, presence of unnecessary ligands, and evaluation of stereochemical and energetic parameters to avoid compromising the overall quality and biological relevance of the structure [44].

### 3.3. Theoretical and Modeling Structures in In Silico Studies

The development of obesity involves multiple factors and metabolic pathways. Establishing possible targets is important for the exploration of new anti-obesity therapies [16]. Despite the rigorous stages preceding the entry of a new drug into the market, the failure rate of drugs that reached the clinical phase in recent decades has been 90%, and even higher when it comes to pre-clinicians [45]. Therefore, the incorporation of methodologies from the field of bioinformatics has emerged as a valuable ally in the screening and design of new candidates by contributing, among other aspects, to the identification of pharmacokinetic characteristics and the early identification of possible toxicity [46].

The existence of 3D structures of therapeutic targets of interest, such as enzymes and receptors, whether deposited in databases or accessible through primary sequence for computational modeling, facilitates the utilization of in silico tools [20]. Employing computational techniques can be a preliminary step, applied to identifying leading candidates, based on criteria like affinity and specificity, which are more interesting in order to proceed to later steps, including synthesis and/or testing to assess the therapeutic potential [44]. Among the studies, the predominance of experimental structures was observed, which were generated by the X-ray crystallography technique (Table 4).

### 3.4. Animal Models Employed in the Validation of In Silico Studies

The animal models used for in vivo validation included various strains of small rodents, encompassing mice or rats. It is noteworthy that among the 12 studies, 10 exclusively used male animals. In animal models, the categorization of obesity induction can be performed in genetic and non-genetic models [47]. Among the selected studies, non-genetic models predominantly prevailed, with obesity induced through high-glycemic and/or high-fat diets. Only one study used a genetic model of induction, employing the ob/ob mice, a monogenic animal model for the induction of obesity by leptin deficiency (Table 3) [43].

Animal models are widely recognized as viable options and established choices for preclinical studies targeting diet-induced obesity. Sexual dysmorphism is an aspect that must be considered in the selection of animal models for studying obesity [48]. Typically, male animals are selected due to their greater susceptibility to obesity-related comorbidities, another advantage is the more rapid and prominent development of obesity in young male animals compared to female animals [48,49]. These models hold the advantage of partially mimicking human obesity through dietary induction. In such models, the interaction between dietary and genetic factors contributes to the development of metabolic disturbances similar to those observed in humans, including insulin resistance. However, a limitation of these models is the absence of standardization across studies [47].

The use of animals In preclinical research is fundamental for the development of new safe drugs [47]. However, the association of in vivo and in vitro studies with bioinformatics techniques can improve research design, thus minimizing the number of animals used in experiments [50], research time, and costs [44].

### 3.5. In Silico-Studied Therapeutic Targets in Obesity

**Figure 3:**
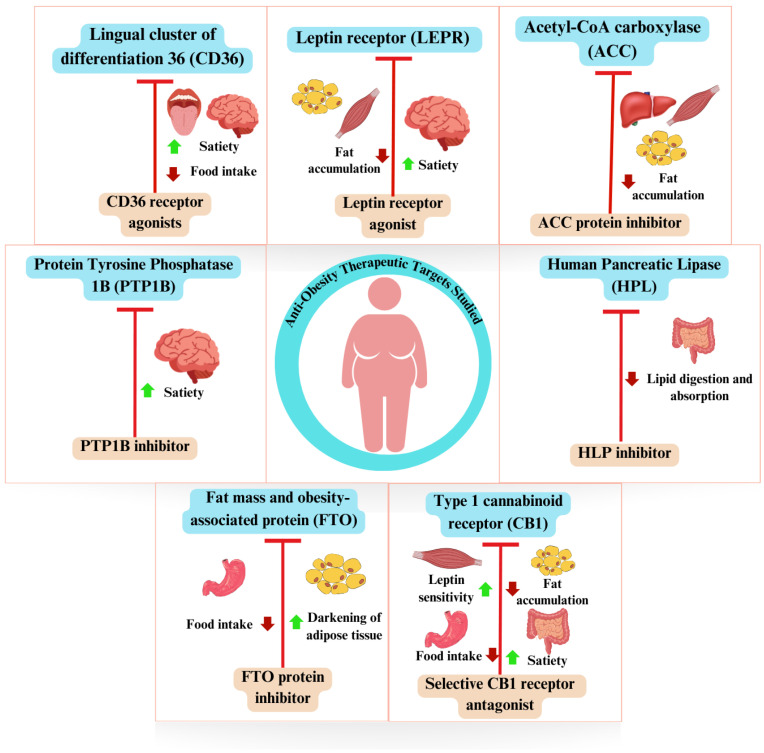
The general diagram of therapeutic targets for obesity studied in silico and reassessed in vivo, and possible metabolic effects of modulation. The upward arrow indicates an increase and the downward arrow indicates a decrease.

The seven therapeutic targets were grouped according to possible mechanisms and outcomes of interest related to their modulation for anti-obesity purposes, aiming to provide a more comprehensive perspective of the treatment process. Figure 3 presents a simplified overview of the potential therapeutic effects associated with modulating the targets identified in this review.

**Figure 4:**
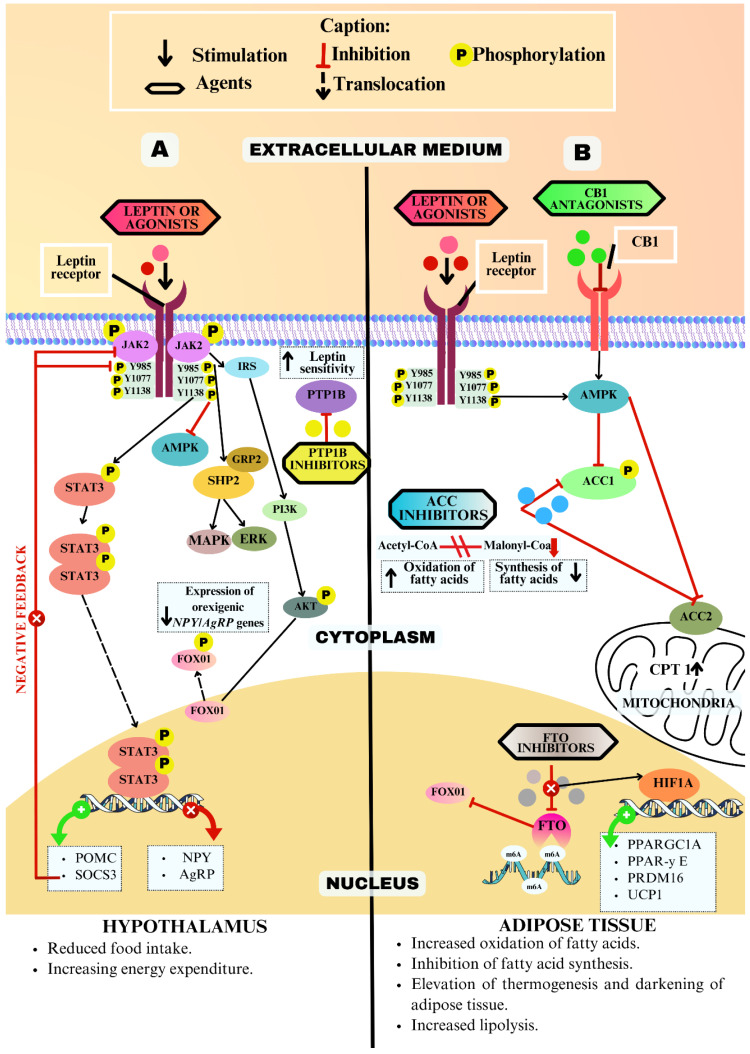
Descriptive diagram of some of the modulations in metabolic pathways associated with the six therapeutic targets of obesity studied in silico, emphasizing the expected modulations in the hypothalamus (**A**) and adipose tissue (**B**). The figure shows the possible therapeutic effects beneficial to body weight control body weight regulation. Legend: Y: tyrosine; P: phosphorylation; FTO: fat mass and obesity-associated protein; STAT3: signal transducer and activator of transcription 3; JAK2: Janus kinase 2; FOXO1: forkhead transcription factor 1; ERK1/2: extracellular signal-regulated kinases 1/2; PTP1B: protein tyrosine phosphatase 1B; AKT: protein kinase B; ACC1: Acetyl-CoA carboxylase 1; ACC2: Acetyl-CoA carboxylase 2. AMPK: AMP-activated protein kinase; NPY: neuropeptide Y; *AGRP*: agouti-related protein gene; *HIF1A:* hypoxia-inducible factor 1 subunit alpha gene; *POMC:* pro-opiomelanocortin gene; CPT I: carnitine palmitoyl transferas1; m6A: N6-methyladenosine; *UCP1:* uncoupling protein 1 gene; MAPK: mitogen-activated protein kinase; *PPAR-Y:* peroxisome proliferator-activated receptor γ gene; SOCS3: suppressor of cytokine signaling-3 gene; SHP2: tyrosine phosphatase 2; PI3K: phosphoinositide 3-kinase and IRS: insulin receptor substrate. Source: Authorship.

**Figure 5:**
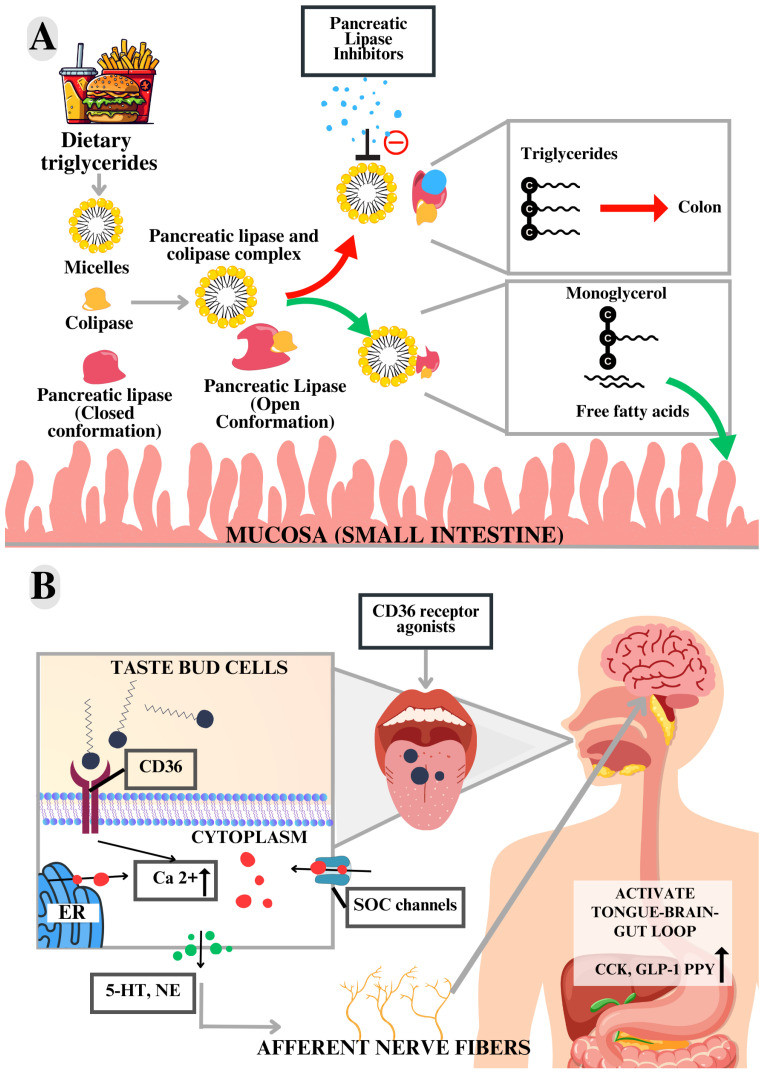
Diagram showing pancreatic lipase modulation in the gastrointestinal tract and CD36 receptor modulation in the oral cavity. (**A**) Diagram of the mechanism of action of Human Pancreatic Lipase (HPL) and the effect of inhibitors on lipid absorption. In the presence of fats and bile salts, in the small intestine, HPL complexes with colipase, which triggers a process of structural modification with the opening of its hydrophobic cap, enabling the anchoring of HPL to the micellar surface and culminating in the hydrolysis of triglycerides and release of monoglycerol and free fatty acids, promoting the absorption of dietary lipids in the presence of HPL inhibitors; hydrolysis of the ester bond of triglycerides is inhibited, resulting in reduced intestinal absorption of fats with a consequent increase in fecal excretion of lipids. (**B**) Basic signal transduction mechanism after interaction of long-chain fatty acids with CD36 present in the taste buds. After the interaction with CD36, there is the release of calcium present in the endoplasmic reticulum and the influx of calcium into the cell through the SOC channels, resulting in the elevation of cytoplasmic calcium and cell depolarization with induction of neurotransmitter exocytosis, activating afferent nerve fibers, and transmitting the information about the stimuli received to the brain. ER: endoplasmic reticulum; SOCs: store-operated calcium channels; 5-HT: serotonin; NE: noradrenalin; GLP-1: glucagon-like peptide-1; PYY: peptide YY; CCK: cholecystokinin. Source: Authorship.

As shown in Figure 4 and Figure 5, the modulation of targets using antagonists, inhibitors, and agonists can occur via metabolic pathways of both central and peripheral action. Therefore, distinct therapeutic agents can trigger responses through the activation of different and/or interconnected metabolic pathways. Detailed information regarding the therapeutic agents can be obtained in Supplementary Table S2.

#### 3.5.1. Leptin Receptor (LEPR)

Leptin is a hormone produced mainly by adipose tissue, which exerts central and peripheral actions via interaction with LEPR [51]. The modulation of LEPR activation may be a therapeutic target for obesity due to the impact on metabolic pathways involved in the control of food intake, lipid metabolism, and energy balance [43,52]. Even with heightened serum levels, individuals with obesity can exhibit leptin resistance, impairing its functionality. Although the mechanisms underlying this resistance are not fully understood, efforts are being made to discover strategies that can restore responsiveness to leptin signals [52].

The interaction between leptin and its receptor in both muscle and adipose tissue triggers the activation of AMP-activated protein kinase (AMPK) [53,54]. Once activated, AMPK phosphorylates both ACC isoforms, thereby suppressing their activity. This inhibition results in a decrease in the concentration of malonyl-CoA, consequently leading to the suppression of fatty acid synthesis and/or increased fatty acid oxidation [55,56]. However, the activation of hypothalamic AMPK stimulates an increase in appetite and a reduction in energy production, so that in the hypothalamus leptin acts by suppressing AMPK activity and contributing to reduced food consumption (Figure 4) [57].

The binding of LEPR also leads to the phosphorylation of tyrosine residues present in the receptor. This subsequently initiates the phosphorylation cascade involving Janus kinase 2 (JAK2) and the signal transducer and activator of transcription 3 (STAT3). The phosphorylated homodimer of STAT3 translocates to the nucleus regulating gene transcription and expression. Thus, the activation of the JAK2/STAT3 pathway modulates the energy balance via the regulation of neuropeptides, increasing the expression of the anorectic pro-opiomelanocortin (POMC), and repressing the expression of the agouti-related protein (AgRP) [58].

Kang et al. [43] used experimental structures of LEPR subdomains (PDB ID: 3V6O e 1I1R) in their in silico study to select possible therapeutic agents. Despite the fact that the extracellular region of LEPR has six functional subdomains [51], the study focused on two specific structures: cytokine receptor II homology domain (CRH2), known for its high affinity for binding to leptin, and the immunoglobulin-like domain (IGD), essential for receptor activation [43]. Docking studies between Ishophloroglucin A and the target subdomains showed interactions with residues Leu471, Tyr472, Leu505, and Leu506 of the active site in the CRH2 structure and with the residue Cys6 of IGD [43].

To verify whether the results obtained in silico are consistent with the biological behavior in vivo, the authors used an experimental model of leptin deficiency (ob/ob mice). The treatment of the animals with IPA promoted a reduction in body weight, a reduction in food consumption, and an improvement in the lipid profile compared to the animals receiving only saline solution (*p* < 0.001). The findings were attributed to the possible activation of the leptin receptor. The hypothesis is reinforced by the increase in brain phosphorylation of proteins that comprise the metabolic pathways associated with LEPR activation, such as JAK2, STAT3, STAT5, and AKT [43]. The result corroborates what was observed by Yu et al. [59], where the administration of tea saponin led to a reduction in body weight and food intake with a hypothalamic increase in phosphorylated STAT3 and *POMC* expression.

Therefore, the exploration of these subdomains through in silico methodologies presents itself as a viable proposition for inclusion within investigations that are oriented towards the screening and/or development of agents capable of facilitating the modulation of the LEPR signaling cascade. It is essential, however, to investigate in vivo to ascertain whether the agent can exert its biological functions in a model of obesity-induced leptin resistance.

#### 3.5.2. Protein Tyrosine Phosphatase 1B (PTP1B)

Another investigated target is PTP1B, the activation of which exerts an adverse influence on the leptin signaling pathway by promoting the JAK2 dephosphorylation (Figure 4) [60]. Given its role in attenuating sensitivity to both leptin and also to insulin, the inhibition of PTP1B’s activity has been the object of study within the context of comorbidities such as obesity and diabetes mellitus [41]. Confronted with the challenge of developing inhibitors with greater selectivity and enhanced cell permeability [41], the study used bioinformatics tools, applying the structural knowledge of the target to design PTP1B inhibitors that could mitigate the limitations of existing inhibitors.

The use of bioinformatics tools in the search for PTP1B inhibitors is due, in part, to the fact that its catalytic site is highly conserved. This creates difficulties for the development of new agents with selectivity against other protein tyrosine phosphatases (PTPs), especially T-cell protein tyrosine phosphatase (TC-PTP) [61]. The significance of selectivity in this context is underscored by the pivotal role played by TC-PTP in immune regulation. Its importance was particularly observed in experiments with knockout mice (TC-PTP−/−). In a previous study, all homozygous animals died between three and four weeks post-birth due to dysfunctions in hematopoiesis and immune function [62].

The active site of PTP1B encompasses highly conserved loops common among protein tyrosine phosphatases (PTPs). These loops include the P, WPD, Q, E, and pTyr loops, alongside a non-conserved secondary site that acts in modulating binding specificity. Within this context, the pivotal residues are Arg24 and Arg254 [41,61]. Thus, inhibitors aimed at the P loop exhibit low specificity, as they tend also to interact with TC-PTP [41].

Ghareb et al. [41] sought to develop double bond inhibitors, designed to interact with the P loop and specific amino acid residues in the B secondary site (Arg24, Ala27, Ser28, Asp29, Phe52, Cys32, Arg254, and Met258), leading to increased selectivity of agents. In their study, the PDB ID 1L8K structure was also used to verify the affinity for the TC-PTP active site and the B site. Thus, upon in vivo administration, the designed agents showed the ability to significantly reduce body weight (*p* ≤ 0.05) [41].

#### 3.5.3. Fat Mass and Obesity-Associated Protein (FTO)

The FTO protein was studied as a possible anti-obesity target due to its role as an RNA demethylase found in various tissues and organs. Its main substrate is N6-methyladenosine (m6A) [63]. The m6A modification in mRNA is more prevalent and abundant in eukaryotic cells [64]. Its impact on many metabolic processes is studied, including the regulation of lipid metabolism [65], so its overexpression has been associated with increased weight and body fat mass [66].

Among the anti-obesity mechanisms, it is possible to increase thermogenesis by stimulating the darkening of adipose tissue. In mice deficient in FTO, there is a reduction in body weight and an increase in thermogenesis. These findings are accompanied by a rise in m6A levels of mRNA of *Hif1a*, a factor that stimulates its translation and positively modulating its expression in adipose tissue [67]. The use of an inhibitor also promoted weight reduction and thermogenesis increasing associated with the downregulation of the FOXO1 transcription factor [68]. In this way, the transcription of thermogenic genes such as *PPARGC1A*, *PPAR-Y*, and *PRDM16* is promoted and the expression of *UCP1* is increased, promoting the darkening of adipose tissue [67,68].

Elekofehint et al. [36] identified phytoconstituents from the aqueous extract of *Annona muricata*, with the aim of assessing their potential as inhibitors of the FTO protein. Through this analysis, hydrogen bonds and hydrophobic interactions were identified, highlighting the crucial role of the amino acid residue Arg96 in the in silico interaction of the two agents (annonaine and annonioside) and the target.

In their subsequent in vivo validation study, Elekofehint et al. [36] observed a reduction in FTO gene expression and an increase in *STAT3* mRNA expression in the pancreas of obese rats treated with the aqueous extract of Annona muricata, when compared to the control group with untreated obesity (*p* < 0.05). However, it is emphasized that additional tests are necessary to ensure aspects related to the safety of use, specificity, and efficiency.

Fajriaty et al. [38] also studied the FTO protein and it was reported in computational studies that one of the most promising compounds, caloxanthone B, formed interactions with the same amino acids as the positive control Orlistat (Tyr 108, Glu 234, and Ser 229). Yaccoubi et al. [37] also tested synthetic thiadiazinone derivatives, highlighting compounds number 16 and 18, which demonstrated interactions with target amino acids Lys216, Asn101, and Leu91. Both studies reported promising effects in the studied obesity models [37,38].

#### 3.5.4. Lingual Cluster of Differentiation 36 (CD36)

Lingual cluster of differentiation 36 (CD36) is a transmembrane protein with a high affinity for long-chain fatty acids found, among other tissues, in taste bud cells (TBC), being explored in pharmacological studies as an anti-obesity target [69]. One of the hypotheses is that the alteration in the sensory perception of fat receptors such as CD36 and G protein-coupled receptor 120 (GPR120), when reduced, may contribute to the development of obesity [70]. Prosérpio et al. [70] observed that individuals with obesity have less sensitivity to fat and have a lower number of fungiform papillae (FP), low oro-detection may imply an increased compensatory intake.

Thus, due to the role of CD36 as a lipid receptor present in the cells of the taste buds, Khan et al. [39] studied the use of non-caloric agonists as possible modulators of the sensory perception of fat and eating behavior (Figure 5). The CD36 structure used in the in silico study was obtained in part from the PDB 5LGD:A, encompassing residues 35–434. The structural predictions were performed through PREDDIMER served as the templates for residues 8–29 and 440–461 [39].

Furthermore, in vivo treatment using the agent NSK-5 resulted in increased levels of intestinal peptides, namely cholecystokinin (CCK), peptide YY (PYY), and glucagon-like peptide-1 (GLP-1) in the systemic circulation. These are peptides considered important for energy homeostasis since they act in signaling satiety and eating behavior. Treated animals showed significantly lower body weight gain and lower food consumption. Therefore, a possible benefit is the contribution to the control of excess body weight present in obesity, by modulating the signaling of important pathways of the tongue–brain–intestine axis associated with satiety [39].

#### 3.5.5. Acetyl-CoA Carboxylase (ACC)

The inhibition of ACC is a therapeutic strategy under study, emerging as a prospective approach for the clinical management of metabolic disorders [71]. In mammals, this enzyme is found in two isoforms, type 1 and type 2 (ACC1 and ACC2), each exhibiting disparate tissue distributions. ACC1 is found in the cytoplasm and predominates in lipogenic tissues (adipose and hepatic tissue). ACC2 is found in the membrane of mitochondria and is more expressed in the heart, muscles, and to a lesser extent the liver [72].

As seen in Figure 4B, ACC has an important role in lipid metabolism, acting in the conversion of Acetyl-CoA to Malonyl-CoA, an indispensable intermediate for the de novo synthesis of fatty acids. Furthermore, Malonyl-CoA is an inhibitor of fatty acid uptake into mitochondria through the inhibition of the carnitine palmitoyl transferase 1 complex (CPT I) [71]. Therefore, the use of ACC inhibitors subsequently reduces the synthesis of long-chain saturated fatty acids, via ACC1 inhibition, and/or increases fatty acid oxidation, via ACC2 inhibition [71,73].

In the study conducted by Chen et al. [42], it was identified that butyrate, a short-chain fatty acid synthesized by the intestinal microbiota, was capable of significantly inhibiting the hepatic production of malonyl-CoA in an animal model (*p* < 0.05), promoting a significant reduction in body weight (*p* < 0.05). In the in silico study, employing an experimental model of the CT domain (PDB ID: 4ASI) of ACC, partial overlap between the butyryl-CoA and malonyl-CoA binding sites with the acetyl-CoA binding site was observed. It is notably that in the body, butyrate is converted to butyryl-CoA. Utilizing molecular docking techniques, the investigation unveiled that binding affinity of butyryl-CoA to the target was similar to that of acetyl-CoA and higher than that of malonyl-CoA. Briefly, it was demonstrated that butyryl-CoA and Malonyl-CoA can competitively bind to ACCase, consequently restoring the balance of lipid metabolism [42].

#### 3.5.6. Type 1 Cannabinoid Receptor (CB1)

The study by Lee et al. [40] explored new type 1 cannabinoid receptor (CB1) antagonists as a possible therapy for obesity, using a theoretical model. Among its actions, it is known that the activation of the type 1 cannabinoid receptor (CB1) contributes to the increase in appetite and increase in body weight, making CB1 antagonists an interesting focus for obesity research [74].

Compound 43c showed promise, demonstrating high selectivity for the CB1 receptor over the CB2 receptor. Additionally, it promoted weight loss of 29.5 ± 1.9% in an animal model of diet-induced obesity compared to untreated animals (*p* < 0.01). Through in silico docking, an interaction was observed with the residue Lys192. These residues are also known to be important for the interaction of the CB1 antagonist drug Rimonabant [75].

The new generation of peripheral CB1 antagonists show promise in controlling body weight, although their mechanisms are not fully understood. However, the evidence suggests increased lipid β-oxidation in adipose tissues due to AMPK activation and heightened carnitine palmitoyl transferase-1 activity (a rate-limiting enzyme for the oxidation of fatty acids) (Figure 4B) [76,77]. In the gastrointestinal tract, the inhibition of CB1 increases the level of cholecystokinin (CCK) [78] and reduces the ghrelin level [79], contributing to the control of food consumption. Moreover, these antagonists could resensitize hypothalamic leptin signaling, offering potential benefits for obesity treatment [80].

The first generation of CB1 receptor antagonists gained notoriety through the drug Rimonabant. Despite success in reducing body weight, the sale of Rimonabant is suspended in the European Union due to side effects [81]. Severe neuropsychiatric repercussions, including depression, anxiety, and suicidal ideation were observed, which were attributed to the ability of these agents to cross the blood–brain barrier and act on CB1 receptors in the CNS [82,83].

To mitigate these side effects, research with new generations of antagonists has concentrated efforts on the development of agents with restricted action on the receptors present in peripheral organs, such as the liver, skeletal muscle, adipose tissue, and pancreas [83]. Continuing the focus on safety, it remains crucial to underscore the necessity for agent specificity. This is particularly important considering that CB1, along with CB2, constitutes the endocannabinoid system [83], but CB2 is mainly expressed in immune cells [75].

#### 3.5.7. Pancreatic Lipase (PL)

HPL was the most explored therapeutic target among the studies included in the research. Coronado-Cáceres et al. [33] administered whole cocoa protein and a high-fat diet in an animal model, resulting in increased fecal fat excretion. An increase in the content of total lipids and triglycerides in the animals’ feces was reported, as well as a percentage reduction in lipid absorption compared to non-treated animals (*p* < 0.05). To evaluate the in vitro inhibition potential of porcine LP, the authors conducted in vitro digestion of cocoa proteins followed by an inhibition assay of porcine LP, obtaining a half-maximal inhibitory concentration (IC50) of 1.4 mg/mL. In the docking analysis, the peptides derived from the simulated hydrolysis of albumin and vicilin were employed. This analysis revealed the presence of Van der Waals bonds and hydrogen bonds between one specific peptide (EEQR) and the HPL enzyme.

In Birari et al. [32], flavonoids were examined both in vivo and in vitro. In the in vitro swine LP inhibition assay, two significant compounds exhibited IC50 values of 7.3 µM and 14.9 µM. These compounds individually interact via hydrogen bonds with amino acid residues in the lid region and catalytic triad (Table 3). A variation in the animals’ weight gain during treatment of 23.2 ± 3.6 g was observed compared to 64.2 ± 0.5 g among untreated animals (*p* < 0.05).

In their study, El-Korany et al. [34] used a secondary metabolite of *Aspergillus oryzae*. They obtained IC50 values of 7.48 μg/mL for dry methanolic fraction and 6.62 μg/mL for kojic acid, the main component of the methanolic fraction used. The methanolic extract administered in vivo led to a significant reduction in body weight, food consumption, and improvement of biochemical parameters (*p* < 0.05).

The study by Yakaiah, Dakshinamoorthi, and Sudha [35] used an ethanolic extract of *Myristica fragrans* seeds for both in vitro and in vivo experiments. The potential of the ethanolic extract to inhibit the activity of swine PL was verified, with IC50 values of 11.35 ± 0.11 μg/mL. In an animal model receiving a cafeteria diet, the administration of the *Myristica fragrans* seed extract significantly increased the level of total fecal lipids (*p* < 0.0001). Moreover, the extract exhibited dose-dependent effect, as evidenced by a reduction in the volume and size of adipocytes. Thus, the authors verified the composition of the extract and proceeded to molecular docking to evaluate the interaction between Tetrahydrofuran, the main compound detected, and the residues Phe77 and Leu153 of HPL.

### 3.6. Limitations of the Study

One of the limitations observed in this systematic review concerns the validation process of in silico studies through reassessment using in vivo experimental models. The in silico assessments presented in the included studies were conducted using isolated substances. However, during in vivo studies, treatments often involved non-isolated or unpurified extracts of the agents under investigation. This approach lacked control over potential interactions between other coexisting compounds, as well as comprehensive data on bioavailability and the possibility of influence of these other compounds or substances in other metabolic pathways not studied.

Considering that the focus of the study is centered on identification of the most promising therapeutic targets studied in silico and reassessed in vivo, it is important to emphasize, as limitations of the present research, the investigation of toxicity and the interaction of the agents investigated in the studies with unintended biological targets. In the future, during the screening of agents for the design of new drugs, such aspects should not be overlooked before proceeding to clinical studies, as they may trigger unforeseen side effects and contribute to the increase in the failure rate of new therapies.

In silico tools, therefore, generate valuable insights into the possible interaction of agents with targets and/or biological effects. Therefore, in the present study, research that carried out in vivo validation was selected, since it is essential to consider the non-complete reproducibility of the complexity of biological systems as a limitation of such tools, and in vivo reevaluation is essential to guarantee the efficacy and safety of new therapies.

## 4. Conclusions

Data from the present study, through systematic review, point to the potential of merging of bioinformatics techniques and in vivo studies as a promising strategy for the investigation of novel treatment possibilities for obesity. Across all the studies included in the systematic review, a consistent pattern emerged: the therapeutic targets that were examined in silico through molecular docking demonstrated effects in vivo that aligned with the anticipated and predicted outcomes from the in silico analyses. This congruence between in silico predictions and in vivo observations bolsters the credibility and potential utility of this integrated approach for advancing obesity treatment research. The combination of in silico, in vitro and in vivo techniques has made possible the faster selective and specific identification and/or design of new agent-specific inhibitors in preclinical study models.

Nonetheless, an imperative to enhance the level of protocol detail is evident in both in silico and in vivo studies. This pertains particularly to the comprehensive presentation of the therapeutic agents employed. These mentioned points are important to ensure the reliability and reproducibility of the studies.

## Figures and Tables

**Table 1:** Elements of the research question, according to the eligibility criteria, based on the problem, exposure, and context (PECo), to answer the following question: What are the therapeutic targets used in the treatment of obesity in in silico studies?

Abbreviation	Descriptor	Elements of the Question
P:	Problem	Therapeutic targets used in the treatment of obesity.
E:	Exposure	Obesity.
Co:	Context	In silico studies

Legend: PECo (P, problem; E, exposure; Co, context).

**Table 2:** Strategies of research equations according to electronic scientific databases aiming to answer the following question: What are the therapeutic targets used in the treatment of obesity in in silico studies?

Database	Search Equation
**PubMed** Selected filter: Title/Abstract	(((therapeutic target[Title/Abstract] OR target[Title/Abstract] OR treatment[Title/Abstract]) AND (Obesity[Title/Abstract] OR Obese[Title/Abstract])) AND (in silico[Title/Abstract] OR computer simulation[Title/Abstract])) AND (molecular dynamic simulation[Title/Abstract] OR molecular dynamic[Title/Abstract] OR molecular docking simulation[Title/Abstract] OR molecular docking[Title/Abstract])
**Science Direct** Selected filter: Title, abstract or author-specified keywords	(obesity OR obese) AND (in silico OR computer simulation OR molecular dynamic simulation OR molecular dynamic OR molecular docking simulation OR molecular docking)
**Scopus** Selected filter: Article title, abstract, keywords	TITLE-ABS-KEY (“therapeutic target” OR target OR treatment) AND TITLE-ABS-KEY (obesity OR obese) AND TITLE-ABS-KEY (“in silico” OR “computer simulation”) AND TITLE-ABS-KEY (“molecular dynamic simulation” OR “molecular dynamic” OR “molecular docking simulation” OR “molecular docking”)
**Embase** Selected filter: Title, abstract or author keywords	(obesity:ti,ab,kw OR obese*:ti,ab,kw) AND (‘in silico’:ti,ab,kw OR ‘computer simulation*’:ti,ab,kw) AND (‘molecular dynamic* simulation*’:ti,ab,kw OR ‘molecular dynamic*’:ti,ab,kw OR ‘molecular docking* simulation*’:ti,ab,kw OR ‘molecular docking*’:ti,ab,kw)
**Web of Science**	((ALL=(Obesity OR Obese*)) AND ALL=(in silico OR computer simulation*)) AND ALL=(molecular dynamic* simulation* OR molecular dynamic* OR molecular docking simulation* OR molecular docking*)
**Virtual Health Library** Selected filter: Title, abstract, subject	(therapeutic* target* OR target* OR treatment*) AND (obesity OR obese*) AND (in silico OR computer simulation*) AND (molecular dynamic* simulation* OR molecular dynamic* OR molecular docking* simulation* OR molecular docking*)

**Table 3:** Characteristics and main outcomes of in silico studies with in vivo reassessment. Abbreviation of terminology used in Table 3 is shown as a footnote ^1^.

Study/ Year/References	Study In Silico		In Vivo Reassessment		Possible Molecular Mechanism of Application
	Methodology	Main Residues in the Interaction Interface of the Most Promising Agent/Main Results of Docking or Molecular Dynamics	Lineages/Diet	Treatment/Effects	
**Human Pancreatic Lipase (HPL)**					
Birari et al., 2011 [32]	Docking Software FlexX	Gly76, Asp79, Thr115, His151, Ser152, Phe215, His263, Arg256 Docking score (Kcal/mol) −23.7 and −21.9	Male Sprague Dawley Rats HF diet	**Isolated agent** ↓ Weight gain and Serum TG and CT	Inhibitor
Coronado-Cáceres et al., 2020 [33]	Docking AutoDockTools vina version 4.5	Asn88, Asn92, Lys239, Arg265, Tyr267, Thr271, Ser333, Asp 331 Affinity (Kcal/mol) −6.5	Male Wistar rats HF diet	**Cocoa Seed Proteins** ↑ Total fecal lipids and fecal TG ↓ Fat absorption rate Ø Body weight and fecal CT	
El-Korany et al., 2020 [34]	Docking Molecular Operating Environment 2015.10 version	Lys80, Asn84, Trp252 Docking score (Kcal/mol) −3.7	Male Sprague Dawley Rats HF diet	**Methanolic fraction** ↓ Body weight, food consumption, serum TG and CT, and liver weight	
Yakaiah et al., 2021 [35]	Docking Autodock4	Phe77 E Leu 153 Binding energy (Kcal/mol): −6.2	Male and female albino rats CD diet	**Ethanol extract** ↑ Total fecal lipids ↓ Volume and size of adipocytes	
**Fat Mass and Obesity-Associated Protein (FTO)**					
Elekofehinti et al., 2020 [36]	Docking Autodock vina	Arg96, Tyr108, Leu109, Val228, His231, Asp233 Binding energy (Kcal/mol) −9.2	Male Wistar rats HF diet	**Aqueous extract** ↓ *FTO* mRNA expression ↑ *STAT3* mRNA expression	Inhibitor
Yaccoubi et al., 2022 [37]	Docking PyRx	Lys216, Leu91 and Asn101 Affinity (Kcal/mol): −11.6 and −10.6	Male Wistar rats HF diet	**Isolated agent** ↓ Hepatic steatosis and volume of adipocytes	
Fajriaty et al., 2023 [38]	Docking/molecular dynamics Autodock version 4.2 and Amber16	Tyr108, Glu 234, Ser 229 Docking—Binding energy (Kcal/mol): −9.74 Molecular dynamics—Free energy (Kcal/mol): −34.21	Rats HF diet	**Ethanol extract** ↓ Weight gain, kidney fat, anal fat, and CT	
**CD36 Receptor**					
Khan et al., 2023 [39]	Docking Quick Vina version 2	Val61, Asn250, Leu251, Lys252, Phe266, Ala267, Ser268, Pro269, Val270, Glu271, Asn275, Asp295, Lys369, Leu371, Asn383, Thr385, Thr387 e Glu418 Docking score (kcal/mol): CD36: −8.4	Male C57BL/6J mice HF diet	**Isolated agent** ↓ Daily fat-rich food intake, body weight gain, body fat mass, liver weight, and mRNA expression of *CD36*, Leptin, SREBP1c, SCD1, FAS, and PPAR-γ, LPS, LDL, and IL-6 blood concentrations ↑ Pancreatic-bile secretion, blood concentrations (GLP-1, CCK, PYY and HDL) Ø Lean body mass, serum TG and size of adipocytes	Agonist
**Type 1 Cannabinoid Receptor (CB1)**					
Lee et al., 2008 [40]	Docking Surflex-dock	Thr197, Asp366, Lys192, Ser383 Docking score: Uninformed	Male C57BL/6J mice WD diet	**Isolated agent** ↓ Body weight	Selective antagonist
**Protein Tyrosine Phosphatase 1B (PTP1B)**					
Ghareb et al., 2019 [41]	Docking Glide version 10.1	Arg24, Arg254, Gln262, Arg47, Asp48, Ala217, Ala27, Ser28, Asp29, Phe52, Cys32, Met258 Docking score (KJ/mol): −5.40 and −4.64	Male Wistar rats HF diet	**Isolated agent:** ↓ Body weight, Homa-IR Index, serum TG, serum CT, and blood glucose and insulin	Inhibitor
**Acetyl-Coa Carboxylase (ACC)**					
Chen et al., 2022 [42]	Docking AutoDock vina version 1.1.2	Uninformed Affinity (Kcal/mol): Result presented in the form of a graph.	Male C57BL/6J mice HF diet	**Isolated agent** ↑ Food consumption, liver ACCase, and serum levels of ACBP, GLP-1, and PYY ↓ Body weight and hepatic Malonyl-CoA	Inhibitor
**Leptin Receptor (LEPR)**					
Kang et al., 2022 [43]	Docking CDOCKER	Leu471, Tyr472, Leu505 e Leu506, Leu530, His467, Ser469, Ser470, Arg615 Binding energy (Kcal/mol)—264.3 Leu2, Leu3, Cys6, Lys29, Lys31, Tyr35, e His 37 Binding energy (Kcal/mol)—776.573	Male C57BL/6J-ob/ob mice Control Diet	**Isolated agent** ↓ Body weight, volume of adipocytes in white adipose tissue, liver lipid droplets, and serum TG and CT ↑ mRNA expression of *4EBP* and *FoxO1*, and phosphorylation of JAK2, STAT3, STAT5, ERK1/2, AKT, and mTOR in the cytoplasm of the hypothalamus.	Agonist

^1^ Footnote:. Amino acids: Asp—aspartic acid; Ala—alanine; Arg—arginine; Asn—asparagine; Cys—cysteine; Phe—phenylalanine; Gly—glycine; Gln—glutamine; His—histidine; Leu—leucine; Lys—lysine; Met—methionine; Ser—serine; Tyr—tyrosine; Thr—threonine; Trp—tryptophan; Val—valine. Symbols: ↑ increase; ↓ decrease; Ø there was no significant effect on the parameter. HF: high-fat diet; CD: cafeteria diet; WD: Western diet; CT: total cholesterol; TG: triglyceride; mRNA: messenger ribonucleic acid; proteína FTO: fat mass and obesity-associated protein; STAT3: signal transducer and activator of transcription 3; STAT5: signal transducer and activator of transcription 5; JAK2: Janus kinase 2; HOMA-IR; homeostasis model assessment; ACBP: Acyl-CoA-binding protein; GLP-1: glucagon-like peptide-1; PYY: peptide YY; *4EBP*: 4E-binding protein; *FOXO1*: forkhead transcription factor 1; ERK1/2: extracellular signal-regulated kinases 1/2; AKT: protein kinase B; mTOR: mammalian target of rapamycin; SGPT: serum glutamic pyruvic transaminase; SGOT: glutamate oxaloacetat transaminase; SREBP1c: transcription factor 1c; SCD1: stearoyl-CoA desaturase-1; FAS: fatty acid synthase; PPAR-Y: peroxisome proliferator-activated receptor-γ. Software FlexX, https://nodepit.com/node/com.biosolveit.flexx.docking.FlexxDockingNodeFactory (accessed on 18 April 2024); Autodock vina, https://vina.scripps.edu/ (accessed on 18 April 2024); PyRx: https://pyrx.sourceforge.io/ (accessed on 18 April 2024); Surflex-dock: https://htpsurflexdock.biocomp.uenf.br/ (accessed on 18 April 2024).

**Table 4:** General information about the structures used in the in silico studies.

Target		Experimental Structure			Theoretical Structure Obtained by Modeling	
	PDB ID	Resolution	Method	Year	Source/Sequence Code	Template Structures
Acetyl-CoA carboxylase (ACC)	4ASI	2.80 Å	X-ray diffraction	2012	NA	NA
Fat mass and obesity-associated protein (FTO)	3LFM	2.50 Å	X-ray diffraction	2010	NA	NA
Protein tyrosine phosphatase 1B (PTP1B)	2F70	2.12 Å	X-ray diffraction	2005	NA	NA
Pancreatic lipase (PL)	1LPB	2.46 Å	X-ray diffraction	1994	NA	NA
Leptin receptor (LEPR) CRH2 subdomain Leptin receptor (LEPR) IGD subdomain	3V6O	1.95 Å	X-ray diffraction	2011	NA	NA
	1I1R	2.40 Å	X-ray diffraction	2001	NA	NA
CD36 Extracellular domain CD36 Transmembrane helices	5LGD	2.07 Å	X-ray diffraction	2016	NA	NA
	NA	NA	NA	NA	UniProt ID: P16671	NA
Type 1 cannabinoid receptor (CB1)	NA	NA	NA	NA	Uniprot ID: P21554	PDB ID: 1F88

Legend: NA: not applicable; PDB ID: structure identification in the PDB database.

## Supplementary Materials

The supporting information can be downloaded at: https://www.mdpi.com/article/10.3390/ijms25094699/s1.
