# Supplementary material for: Anti-Obesity Therapeutic Targets Studied In Silico and In Vivo: A Systematic Review
Source: Int J Mol Sci. 2024 Apr 25;25(9):4699. doi: 10.3390/ijms25094699 (PMC11083175; doi:10.3390/ijms25094699)
Supplement: Supplementary file 1 [file ijms-25-04699-s001.zip › Table S1.pdf]

Table S1 – Form for assessing the risk of bias in in silico studies.

Bias Domain	Issue	Low Risk of Bias	High Risk of Bias	Unclear Risk of Bias
Ligand selection	Ligand filtering	Should be performed	Did not applied	No data
Ligands optimization	Ionization assessment	The ligands were ionized according to pKa and pH values of media	The research was performed without reference to pKa values of ligands and pH values of media	No data
	Generation of energetically possible conformations	Should be performed	Generation was performed without reference to potential energy calculation	No data
Target selection	Resolution of protein structure	Not more than 2.5 Å	More than 2.5 Å	No data
	Method of protein target structure obtaining	NMR spectroscopy or X-ray crystallography	Cryogenic electron microscopy or modeling	No data
Target optimization	Control of histidine protonation	Should be performed	The structure of target did not reference biological conditions	No data
	Protonation of amino acids after X-ray crystallography or cryogenic electron microscopy	Should be performed	The structure of target did not reference biological conditions	No data
	Addition of missing residues and side chains after X-ray crystallography or cryogenic electron microscopy	Should be performed	Was performed without special tools	No data
	Addition of metals	Should be performed	The structure of target did not reference biological conditions	No data
Docking	*Molecular docking software*	Glide, GOLD	AutoDock, DOCK, FlexX	No data
Results assessment	Visual control	Should be performed	Structure defects were observed	No data
	Re-docking	Should be performed	The RMSD value is too high compared with the initial structure	No data
	Verification of docking results by in vitro study *in vitro* ou *in vivo*	Binding constant should be determined or performing an in vivo study.	The quantitative calculations were not performed	No data

Source: Adapted from Taldaev and collaborators by the author (22).
