# Supplementary material for: Anti-Obesity Therapeutic Targets Studied In Silico and In Vivo: A Systematic Review
Source: Int J Mol Sci. 2024 Apr 25;25(9):4699. doi: 10.3390/ijms25094699 (PMC11083175; doi:10.3390/ijms25094699)
Supplement: Supplementary file 1 [file ijms-25-04699-s001.zip › Table S2.pdf]

Table S2. Main therapeutic agents employed in in silico studies along with their respective chemical structures and/or formulas.

Study/ Year/ References	Class/Source	Main therapeutic agents used within in silico studies
Birari *et al* 2011., [32]	Flavonoids, Roots of *Glycyrrhiza glabra*	Isoliquiritigenin (7) [a] and Licuroside (10)[a]:<br>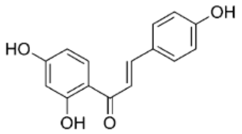<br>7<br>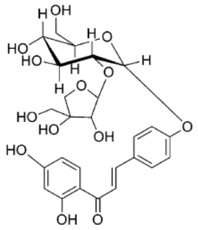<br>10
Coronado-Cáceres *et al*., 2020 [33]	Peptides, Seeds of *Theobroma cacao L*	Cocoa (*Theobroma cacao L.*) peptide EEQR [a]
El-Korany *et al*., 2020 [34]	Organic acid, *Aspergillus oryzae*	Kojic acid[b]
Yakaiah *et al*., 2021 [35]	Heterocyclic Organic Compound, Seeds of *Myristica fragrans*	Tetrahydrofuran[b]
Elekofehinti *et al*., 2020 [36]	Alkaloids, *Annona muricata* (Annonaceae)	Annonioside[b] and Annonaine[b]
Yaccoubi *et al*., 2022 [37]	Tiadiazinona derivative, synthetic	Bis-fused thiadiazinones 16 [a] and 18[a]<br>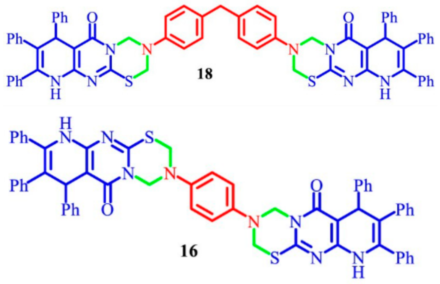
Fajriaty *et al*., 2023 [38]	Phytochemicals, *Calophyllum soulattri*	Caloxanthone B[a]<br>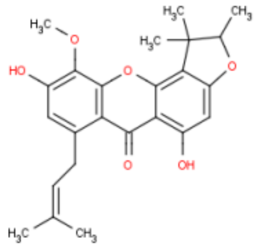
Khan *et al*., 2023 [39]	Linoleic acid derivatives, *synthetic*	NKS-5 [a]: (9Z,12Z)-(3-methyloxetan-3-yl) methyl octadeca-9,12-dienoate<br>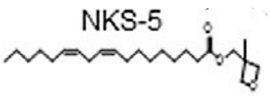

Lee *et al.*, 2008 [40]	Biarylpyrazolyl oxadiazole analogues, synthetic	**43c**[a]: 2-(4-((1H-1,2,4-triazol-1-yl)methyl)-5-(4-bromophenyl)-1-(2,4-dichlorophenyl)-1H-pyrazol-3-yl)-5-(1-(trifluoromethyl)cyclopropyl)-1,3,4-oxadiazole
Ghareb *et al.*, 2019 [41]	**Benzenesulfonamide derivatives, synthetic**	MS-13 [a] and MS-14 [a]: 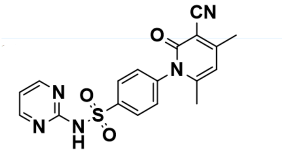 **MSE-13** 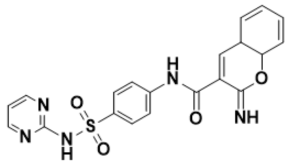 **MSE-14**
Chen *et al.*, 2022 [42]	Short chain fatty acids (SCFAs), bacterial fiber fermentation	Butyrate[b]
Kang et al 2022 [43]	Florotanins, Algae *Ishige okamurae (Yendo*)	Ishophloroglucin A[a] 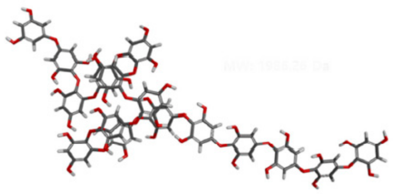

[a] Chemical structure and/or formula obtained in the original article

[b] Chemical structure and/or formula not obtained in the original article
